# Tissue Engineering Chambers: Potential Clinical Uses and Limitations

**DOI:** 10.1016/j.ebiom.2016.03.010

**Published:** 2016-03-12

**Authors:** Roger K. Khouri, Frances M. Walocko

**Affiliations:** University of Michigan Medical School, Ann Arbor, MI, USA

Oxygen delivery is the greatest limiting factor to large-volume tissue engineering. Regenerating post-mastectomy breast tissue requires avascular adipose tissue to be transferred as thin “micro-ribbons” to avoid central necrosis (Khouri et al., 2014). Similarly, scaffold-based constructs lack the central vascularity necessary for large-volume tissue regeneration (Post et al., 2013). The tissue engineering chamber (TEC) induces the recipient bed to regenerate fully vascularized tissue *in vivo*, thereby providing the oxygen delivery necessary to support large-scale tissue regeneration. In the above article (Morrison et al., 2016), Morrison and colleagues describe the first successful use of TEC to generate well-vascularized, large-volume human adipose tissue *in vivo*. While TEC-based constructs do not have the same ability as scaffold-based constructs to control the internal structures of regenerated tissues, they still have potential utility in certain clinical scenarios.

Adipose tissue is an ideal model for *in vivo* soft tissue regeneration because its function does not depend on tissue microanatomy or organ gross anatomy. However, adipose tissue regeneration with TEC lacks major clinical utility because, with proper technique and recipient site preparation, large-volume autogenous adipose tissue transfers can already safely be performed for reconstruction of breasts and other soft tissue defects (Khouri et al., 2015, Khouri et al., 2013). Alternatively, endocrine tissue regeneration has no current clinical substitute, and endocrine functions do not depend on anatomy; a thin sheet of functioning endocrine tissue placed anywhere in the body could replace damaged native tissue. Morrison contributed to a study that transplanted pancreatic islets into TECs in diabetic mice, which resulted in significantly improved glycemic control (Hussey et al., 2009). Reproducing these findings in humans would have an enormous clinical impact given the prevalence of diabetes mellitus type 1 and cases of diabetes mellitus type 2 that require exogenous insulin.

Morrison has also contributed to other rodent TEC studies that have successfully regenerated large volumes of functioning pituitary (Lepore et al., 2007), thymic (Seach et al., 2010), hepatic (Forster et al., 2011), and cardiac tissue (Morritt et al., 2007). While regeneration of any of these tissues in a human model would have important clinical uses, the inability to control the internal structure of the regenerated tissues still limits TEC from regenerating fully functioning non-endocrine organs. For example, Forster and colleagues cultured liver progenitor cells (LPCs) to form three-dimensional clusters within a matrix before implantation in a chronic liver injury model using TEC (Forster et al., 2011). LPCs differentiated into hepatocytes with the ability to perform hepatocyte-specific metabolism and biosynthesis but without the ductal drainage system necessary for whole organ regeneration.

Whole organ regeneration would require the vascularity associated with TEC and the internal anatomy associated with cell scaffold-based constructs. An intricate combination of TEC and a cell-scaffold based construct might create a solution; however, this concept seems well ahead of our current capabilities given the widely varied results Morrison and colleagues obtained using TEC in humans.

Morrison and colleagues should be commended for their novel work. While these findings cannot yet impact clinical practice, they should encourage researchers to continue making strides towards translating tissue engineering breakthroughs to the clinical arena. TEC clearly has great potential for regeneration of human tissues, but before advancing any further, TEC must demonstrate the ability to consistently produce predictable outcomes.

## Disclosures

The authors have no conflicts of interest to disclose.
